# Sterilization characteristics of narrow tubing by nitrogen oxides generated in atmospheric pressure air plasma

**DOI:** 10.1038/s41598-023-34243-3

**Published:** 2023-04-28

**Authors:** Reona Muto, Nobuya Hayashi

**Affiliations:** 1 Interdisciplinary Graduate School of Engineering Sciences, Department of Advanced Energy Engineering Science, Kyushu University, Fukuoka, 816-8580 Japan; 2 International Research Center for Space and Planetary Environmental Science, Kyushu University, Fukuoka, 819-0395 Japan

**Keywords:** Energy science and technology, Engineering

## Abstract

The sterilization characteristics of active species generated by an atmospheric dielectric barrier discharge plasma using air and oxygen at the inner surface of silicone tubing were investigated. A dielectric barrier discharge torch plasma device was installed at one end of the tube and generated long-lived active species that flowed into the tube. A strip-type biological indicator with a 10^5^-cell bacterial spore was placed at the opposite end of the 60 cm tube. Sterilization was completed within 30 min by active particles generated from the air plasma. The main factors contributing to the sterilization by air plasma were HNO_3_ and N_2_O_5_. When organic materials (keratin, aspartic acid, and dipicolinic acid) reflecting components of the bacterial spore, were treated by the sterilization procedure there was little effect on dipicolinic acid. Keratin was oxidized by ozone and NO_x_ generated from the oxygen and air plasmas, respectively. Aspartic acid underwent little change in composition from ozone generated from the oxygen plasma, whereas nitro (NO_2_), nitroso (NO), and aldehyde (CHO) groups were formed from ozone and NO_x_ generated from the air plasma.

## Introduction

Sterilization of medical equipment is an important procedure in hospitals facilitating reuse of equipment. Medical plastic tubing has a long and narrow shape and is difficult to sterilize. Thus, owing to its frequent use, medical tubing is consumed in large quantities. Although it would be desirable to sterilize and reuse tubing to reduce healthcare costs, there are no effective sterilization methods available. Reuse of medical equipment is important even in outer space where the amount of equipment to be transported is limited. Sterilization methods currently used in medical practice that are applicable to sterilization of tubing include ethylene oxide gas (EOG) sterilization and hydrogen peroxide (H_2_O_2_) sterilization. However, these chemicals are toxic and relatively stable, leaving residue that is difficult to remove from narrow plastic tubing^1,2^. Typically, approximately 1 day is required to remove EOG from medical devices following sterilization. Residual toxic and/or carcinogenic chemicals may endanger patients, sterilization operators, and medical professionals. Recently, plasma sterilization methods have been studied owing to potential for low toxicity and reduced sterilization times^2–6^. Although many plasma sterilization methods been applied to plastic tubing, effective sterilization methods have yet to be developed because of heat and damage caused to the tubing material^7–10^.

When an electrical discharge occurs in air, ozone (O_3_)^11–14^ and nitrogen oxides (NO_x_)^15–17^, such as nitrogen monoxide (NO), nitrogen dioxide (NO_2_), and dinitrogen pentoxide (N_2_O_5_), are obtained at atmospheric pressure. Short-lived radicals such as NO_3_ and active oxygen species derived from NO_x_ and ozone can also be generated. These nitrogen species and active oxygen species are highly reactive and undergo decomposition or modify biomaterials, such as proteins, amino acids and DNA. Notably, these stable reactive species are generated in a plasma at room temperature. Therefore, sterilization by ozone and NO_x_ is unlikely to induce thermal deterioration or surface damage to plastic tubing.

In recent years, attempts have been made to sterilize the inner wall of long narrow tubes with oxygen plasmas^10^; however, these approaches have required long processing times. The combination of an oxygen plasma with ultraviolet (UV) light irradiation has achieved sterilization of the inner walls of long narrow tubes in a relatively short time. However, any obstruction of the UV light source may limit this sterilization approach. In this study, sterilization of narrow tubes was investigated based on long-lived active species generated from an atmospheric dielectric barrier discharge (DBD) torch plasma using oxygen gas and air as feed gases. The effects of the plasma sterilization on organic materials including bacterial spores were investigated. This research demonstrates that both the sterilization of the inner wall of a long narrow tube with sufficient length for medical use and sufficient material compatibility can be achieved by the plasma using oxygen gas and air, and the particle species that contribute to the sterilization were investigated. Obtained results will greatly contribute to the demonstration of a long narrow tube inner wall sterilization method.

## Experimental methods

### Measurement of active species

**Figure 1:**
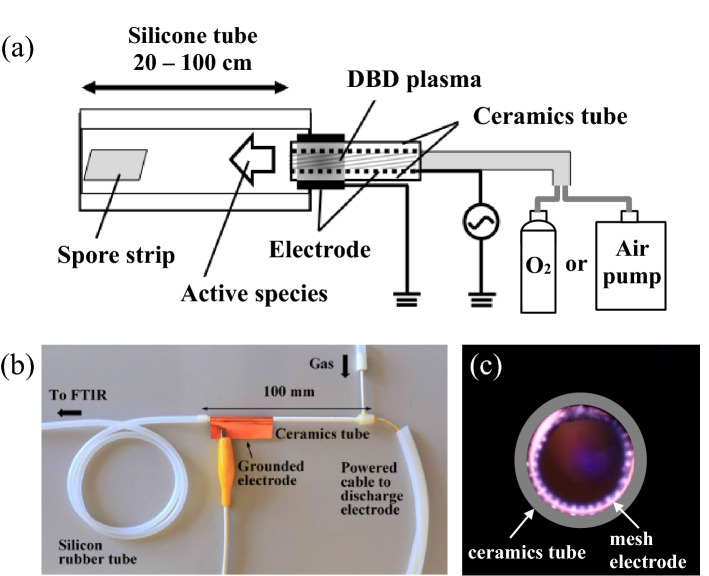
Schematic diagram of experimental apparatus (**a**), a photograph of DBD torch plasma device (**b**) and an axial view of plasma inside the ceramic tube (**c**).

Figure 1a,b show the schematic diagram of the experimental apparatus and the photograph of DBD torch plasma device, respectively. The dielectric barrier discharge (DBD) torch plasma device for the plasma production was installed at the end of the sample silicone tube to be sterilized. The ceramics tube for the DBD torch plasma generation has the dimension of *ϕ* 3.0 in outer diameter, *ϕ* 3.0 in inner diameter and 100 mm in length. Stainless mesh with 50 mesh/inch, 0.23 mm in thickness is wounded inner surface of the ceramics tube as the discharge electrode. When a high voltage and a high frequency were applied to a mesh type electrode set inside the ceramics tube, a DBD occurred around the mesh electrode, as shown in Fig. 1c. Light emission by the discharge is observed in the vicinity of the mesh type electrode. The voltage source used in this study was a capacitively coupled AC high voltage power supply with the maximum output power is 100 W (Logy Electric, LHV-10AC). The voltage applied to the electrodes was 8.1 kV_pk-pk_, and the frequency of the applied voltage was 9.8 kHz. The feed gases for the plasma generation were oxygen gas (99.99% purity) or ambient air. The oxygen gas was supplied from a gas cylinder, and ambient air was introduced into the plasma torch and the sample silicone tube using a small air compressor. The gas flow rate was maintained at 0.4 L/min. By introducing oxygen gas or air into the DBD torch plasma device, active species were produced in the air and oxygen plasmas. The active species flowed from the DBD torch plasma device under the gas flow and were transported inside the sample silicone tubing and sterilized its inner surface. When ambient air is used for plasma production and air humidity is not controlled, water absorbed in the ceramics dielectric of barrier discharge electrode generally tends to extinguish or weaken the discharge. The ceramic dielectric used for the surface barrier discharge is effectively heated by the collision of high-energy particles during the discharge, and the water in the dielectric evaporates several minute after the discharge initiation^18^. After that, the discharge in humid condition recovers and is almost the same as discharge in dry air^19^. In this experiment, the sterilization process starts after 30 min warm-up from the discharge initiation.

To identify active species responsible for microbial inactivation, the compositions of the active species in the gas flow exhaust from the sample silicone tube were investigated. Concentrations of O_3_ and NO_2_, which are relatively long-lived species contained in the gas flow generated from the air plasma, were measured by gas detection tubes. Other gas species in the flow were analyzed by Fourier transform infrared spectroscopy (FTIR). FTIR spectra were measured for the gas flow through the plasma source (torch), for the silicone tube to be sterilized, and from a connected FTIR gas cell. The gas flowed from the silicone tube into the FTIR gas measurement cell (capacity 50 mL) at a flow rate of approximately 0.4 L/min; hence, the gas in the cell was replaced within 1 min. The time required to measure the FTIR spectrum was approximately 30 s. Therefore, the time required to analyze gas ejected from the tube was approximately 2 min. This time is sufficiently short compared with the half-lives of HNO_3_ and N_2_O_5_ (both several hours) such that any decomposition of these species was negligible. The measurements were performed 10 min after the start of plasma generation. Throughout the sterilization process, the plasma was constantly generated and the ozone concentration, which is approximately proportional to the density of the generated plasma, reached a constant value. Therefore, the amounts of generated gas species, such as O_3_, NO_2_, HNO_3_, and N_2_O_5_, became constant over time. The FTIR spectra were measured eight times and an average value was obtained. The variation in the spectra of O_3_, NO_2_, HNO_3_, and N_2_O_5_ was less than 1%.

In both air and oxygen plasmas, active oxygen species are the key components that contribute to sterilization. To measure the amounts of active oxygen species at the end of the sample narrow tube, a chemical indicator (CI) for detecting atomic oxygen, was used. The CI was a sheet coated with a phthalocyanine pigment, which changed from purple to green depending on its degree of contact with the active oxygen species. The CI was placed at the target in Fig. 1 where samples were placed for the sterilization treatment. The rate of color change of the CI to green was determined as the numerical G (green) value from a scanner based on the color model (RGB). To quantify the amount of active oxygen species, ΔG indicates the difference in G values before and after processing. Relationships among the tube length and the reach of active oxygen and differences between the air and oxygen plasmas were evaluated.

$$\Delta {\text{G}} = \left\{ {\left( {{\text{G}}\;{\text{value}}\;{\text{after}}\;{\text{treatment}}} \right) - \left( {{\text{G}}\;{\text{value}}\;{\text{before}}\;{\text{treatment}}} \right)} \right\} \times {1}00/\left( {{\text{G}}\;{\text{value}}\;{\text{before}}\;{\text{treatment}}} \right)$$

### Sterilization characteristics

A DBD torch plasma device was installed at the end of a sample silicone tube with an inner diameter of 4 mm. To evaluate the sterilization characteristics of this method, a strip-type biological indicator (BI) with bacterial spores (*G. stearothermophilus*, approximately 10^5^ cells) was placed at the end of the tube opposite to the plasma device. Irradiation times ranging from 15 to 180 min were examined. Three BIs were used for each irradiation condition. The treated BI was cultured in tryptic soy broth at 58 °C. The effectiveness of sterilization was determined based on the change in color of the pH indicator in the culture solution after cultivation for 24 h. The distance between the plasma device and BI inside the tube varied from 20 to 100 cm.

### Evaluation of material compatibility

To investigate the material deterioration caused by irradiation with the active species generated from the plasmas, changes in chemical bonds on the surface of polyethylene terephthalate (PET), polyvinyl chloride (PVC), and silicone were measured by ATR-FTIR. Variation of the heights in typical peaks of the FTIR spectrum of each material were used to infer decomposition or modification of the surface structure. The rate of compositional change was calculated by comparing the heights of the peaks of untreated and sterilized material samples. Spectra were recorded over the wavelength range of 4000–500 cm^−1^ at 2 cm^−1^ spectral resolution. Background spectra were measured in ambient air. To prepare samples for material compatibility measurements, a PET or PVC strip was cut to 4 mm in width and placed in a 60 cm long tube at the same position as the BI and was subjected to the sterilization. For the silicone sample, the end of the treated 60 cm silicone tube was cut to obtain a piece with a width of 2 mm. These samples were sterilized for 0, 2, 4, 8, and 10 h.

### Investigation of spore inactivation mechanism

To investigate irradiation-induced changes in the chemical composition of organic materials contaminated with bacterial spores, samples were prepared with pure powdered reagents (i.e., keratin, aspartic acid, glutamic acid, dipicolinic acid) adhered to calcium fluoride plates. These chemicals are components of organic matter contained in bacterial spores. These organic samples were irradiated with active species, and changes in the chemical composition of materials were measured by FTIR. Wool keratin was selected as keratin reagent because of its similarity to the keratin-like proteins of bacterial spores^20,21^.

## Results and discussion

### Generation of active species

**Figure 2:**
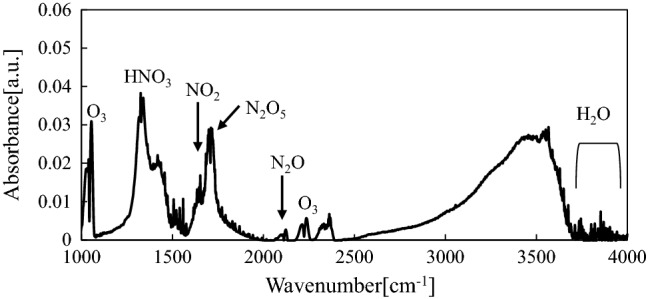
Typical IR spectrum of exhaust gas generated from air plasma.

To specify particles of the sterilization factor generated in plasmas, FTIR spectra of the exhaust gas from the tube were measured. In the case of the oxygen plasma, ozone was generated and transported into the sample tube. Ozone dissociates into oxygen molecules and an active oxygen species, singlet oxygen atoms O(^1^D), by autolysis. The sterilization of the long narrow tube subjected to the oxygen plasma was attributed mainly to the ozone-derived O(^1^D), which has strong oxidation potential and a relatively long lifetime. In the case of air plasma, nitrogen oxides are produced and interact with ozone^22^. Peaks attributed to HNO_3_ at 1325 cm^−1^, NO_2_ at 1627 cm^−1^, N_2_O_5_ at 1718 cm^−1^, and N_2_O at 2210 and 2236 cm^−1^ were observed as shown in Fig. 2. The NO_2_ concentration measured by the gas detection tubes was less than 25 ppm, and NO was not detected. Ozone was also observed in the FTIR spectra at approximately 1030 and 1055 cm^−1^. The ozone concentration ranged from 100 to 280 ppm in the gas detection tubes regardless of the tube length. In the case of the oxygen plasma, the ozone concentration ranged from 500 to 760 ppm. Therefore, ozone and NO_x_ contributed to sterilization inside the tubing. Compounds having relatively long lifetimes and high reactivity generated from ozone and NO_x_ contributed to the sterilization.

**Figure 3:**
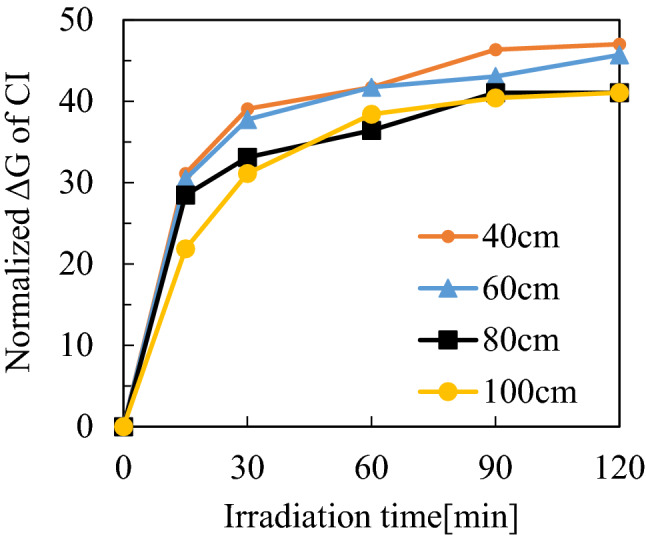
CI discoloration rate with respect to tube length and air plasma treatment time.

**Figure 4:**
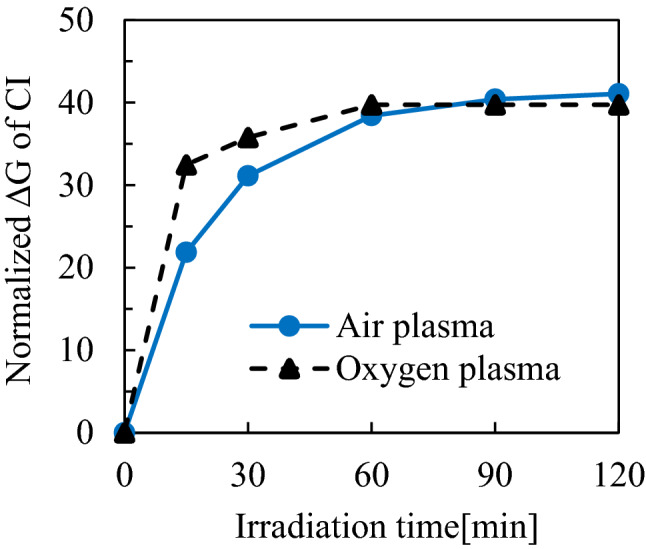
CI discoloration rate of air plasma and oxygen plasma for a tube of 100 cm.

To confirm the amount of active oxygen species flowing in the sample tube, the relationship between the irradiation period and the discoloration rate of CIs was investigated. In this study, O(^1^D) derived from ozone was presumed to be the main active oxygen species that changed the color of the CI. As shown in Fig. 3, the amount of the O(^1^D) derived from the air plasma increased with the irradiation period for approximately 60 min and thereafter increased more gradually over the irradiation period. Also, the amount of ozone detected depended on the tube length, and the difference in the rate of the color change was within approximately 10% between 40–60 cm and 80–100 cm. The amounts of active oxygen species were compared between the oxygen plasma and air plasma. As shown in Fig. 4, inside a tube with a length of 100 cm more O(^1^D) was generated from the oxygen plasma than from the air plasma.

### Sterilization characteristics and factors

The sterilization characteristics of the BIs were evaluated. Table 1 shows the sterilization results of BIs set in the sample tube with a length of 20 cm based on air and oxygen plasmas. The results are listed in Tables 1 and 2. In the case of the oxygen plasma, sterilization of the BI was successful for only one sample subjected to the 30 min treatment. Conversely, sterilization was successful for all conditions in the case of the air plasma. Table 2 shows sterilization results using air plasma for tubes with lengths of 40 to 100 cm. The tube 60 cm in length was completely sterilized by air plasma within 30 min. Although there were some fluctuations, the relationship between ΔG and the sterilization probability of the BI with different tube lengths followed a similar tendency, as shown in Fig. 4 and Table 2. Therefore, active oxygen species that reached the end of the sample tube may also be capable of inducing sterilization.

**Table 1:** BI sterilization results for oxygen and air plasmas in a tube of 20 cm.

	30 min	60 min	90 min	120 min
Oxygen	1/3	0/3	0/3	0/3
Air	3/3	3/3	3/3	3/3

**Table 2:** BI sterilization results for air plasma in tubes ranging from 40 to 100 cm.

	40 cm	60 cm	80 cm	100 cm
15 min	0/3	0/3	–	–
30 min	3/3	3/3	1/3	0/3
60 min	3/3	3/3	2/3	2/5
120 min	–	–	2/3	2/5
180 min	–	–	–	2/5

The ozone density did not depend on tube length. Although the oxygen plasma generated a high ozone density, the sterilization rate of the oxygen plasma was much lower. Hence, the difference of the sterilization rate between the air and oxygen plasmas was not determined by the ozone density alone. Furthermore, there was no decrease in the ozone density, even when the ozone reached the end of the longer tube. Thus, most of the ozone was not dissociated and flowed out of the tube in the form of ozone. Therefore, the density of O(^1^D) formed by autolysis of ozone in the tubes was relatively small. The O(^1^D) is likely not produced by natural self-decomposition of ozone, but rather through its interactions with reactive surfaces, such as the CI and bacterial spores in the BI. The oxidation reaction likely caused discoloration of the CI and sterilization of the BI.

In this experiment, at the end of the 100 cm tube, the air plasma produced ozone at a concentration of 100–280 ppm and a smaller amount of active oxygen than did the oxygen plasma, as shown in Fig. 4. According to previous studies, sterilization of *G. stearothermophilus* may be achieved by ozone at a concentration of 250 ppm with a treatment period of 100 min^23^. Thus, if ozone is the only factor contributing to sterilization in the air plasma, more than 100 min would be needed for complete sterilization. Therefore, the ozone and O(^1^D) were not the only factors contributing to sterilization. Another study has reported that NO_2_ at 5,000 ppm sterilized bacterial spores within 30 min^24^. Because the concentration of NO_2_ in this experiment was approximately 25 ppm and significantly lower than that in the above previous work, it may be inferred that NO_2_ did not have a major contribution to the sterilization.

**Figure 5:**
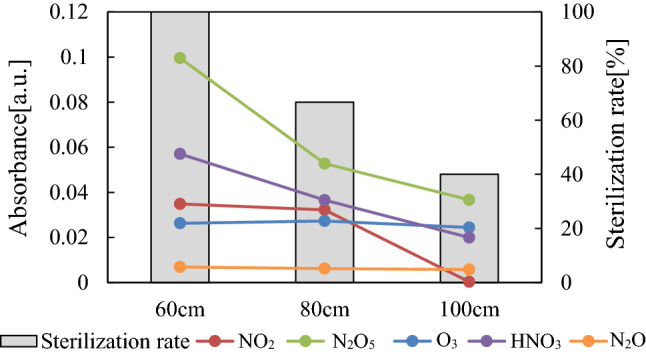
Associations between absorbance of gas components and sterilization rate by air plasma and tube length.

The observed sterilization effect of the air plasma was higher than that of the oxygen plasma. The difference in the sterilization rates of the air plasma and the oxygen plasma may be attributed to other NO_x_ species. Figure 5 compares the peak height of each component and the success rate of sterilization for 60 and 100 cm tubes. In terms of ozone, there was no notably difference between the 60 and 100 cm tubes, which is consistent with the fact that the ozone concentration did not change over the tube length. Conversely, the peaks of NOx were higher for the 60 cm than for the 100 cm tubes. Thus, the amounts of HNO_3_ and N_2_O_5_, which were not measured by the detector tubes, were likely to be higher for the 60 cm tube. HNO_3_ is strongly oxidizing and may behave as a sterilization factor. N_2_O is known to very unreactive with a long lifetime in air. Additionally, N_2_O decomposes in the stratosphere, as shown by the following equations^25^.

1 $${\text{N}}_{{2}} {\text{O}} + {\text{h}}\nu \to {\text{N}}_{{2}} + {\text{O}}\left( {^{{1}} {\text{D}}} \right) \to \lambda < {\text{337 nm}}$$

2 $${\text{N}}_{{2}} + {\text{O}}\left( {^{{1}} {\text{S}}} \right) \to \lambda < {21}0\;{\text{nm}}$$

3 $${\text{NO}} + {\text{N}}\left( {*{\text{S}}} \right) \to \lambda < {25}0\;{\text{nm}}$$

4 $${\text{N}}_{{2}} {\text{O}} + {\text{O}}\left( {^{{1}} {\text{D}}} \right) \to {\text{N}}_{{2}} + {\text{O}}_{{2}}$$

5 $$\to {\text{2 NO}}$$

Because NO was not observed by the detector tube or by FTIR it was unlikely that the decomposition of N_2_O had a role in the sterilization or that N_2_O reacted with the sterilized objects in this study. N_2_O_5_ is related to NO_3_, as shown in Eqs. (6)–(9). NO_3_ is strongly oxidating and an unstable and highly reactive molecule; however, it does not have an unpaired electron, and its reactivity is not as high as that of an OH radical. Therefore, it is conceivable that NO_3_ may have a longer lifetime than ions or atomic oxygen and thus pass through the sample tube. Furthermore, it is possible that the reaction of NO_x_ and ozone in the gas flow from the air plasma might produce NO_3_ instantaneously around the BI, and the NO_3_ may act on bacteria by oxidization and/or and nitration. Reaction (9) refers to thermal decomposition, which has a decomposition rate less than 12% below 40 °C^26^. There was a small amount of NO_3_ at equilibrium with the presence of N_2_O_5_ under the conditions of the experiment. Therefore, NO_3_ may be inferred to contribute to sterilization of the tube in this study.

6 $${\text{NO}}_{{2}} + {\text{O}}_{{3}} \to {\text{NO}}_{{3}} + {\text{O}}_{{2}}$$

7 $${\text{NO}}_{{3}} + {\text{NO}} \to {\text{2NO}}_{{2}}$$

8 $${\text{NO}}_{{2}} + {\text{NO}}_{{3}} + {\text{M}} \to {\text{N}}_{{2}} {\text{O}}_{{5}} + {\text{M}}$$

9 $${\text{N}}_{{2}} {\text{O}}_{{5}} \to {\text{NO}}_{{2}} + {\text{NO}}_{{3}}$$

Additionally, the following gaseous components were estimated to be formed secondarily from the active gas qualitatively analyzed by FTIR. As shown in Fig. 2, the active species included water, and reaction products of NO_3_, O_3_, HNO_3_, N_2_O_5_, NO_2_, and H_2_O were considered to secondarily produce active gas components with sterilizing effects, which were not observed in FTIR spectra. Water in a liquid state might also have been present in the air flow owing to condensation caused by the temperature difference between the discharge region (approximately 60 °C) and the tube (approximately 25 °C). Therefore, active species produced by chemical reactions involving water in liquid state (l, aq) might also be inferred to act as sterilization factors, in addition to the gas components, as shown reaction (10)–(14)^14,27^, (15)–(20)^28^ and (21)–(23)^29^. Among the potential active species, peroxynitrite (ONOO^−^) is capable of being nitrosated (i.e., undergoing addition of NO groups), oxidization of SH groups, nitrosation of NH_2_ groups (i.e., conversion of NH_2_ to NO), oxidization of bacterial proteins and lipids, oxidization and nitrosation of DNA components, and damaging DNA by hydrogen withdrawal from ribosomes^30,31^. Nitrite (HONO) has similar effects on organic materials to HNO_3_^32^, suggesting that ONOO^−^ and HONO may be sterilizing factors.

10 $${\text{O}}_{{3}} + {\text{H}}_{{2}} {\text{O}} \to {\text{2 HO}}* + {\text{O}}_{{2}}$$

11 $${\text{O}}_{{3}} + {\text{OH}}^{ - } \to *{\text{O}}_{2}^{ - } + {\text{HO}}_{2}^{*}$$

12 $${\text{O}}_{{3}} + {\text{HO}}* \to {\text{O}}_{{2}} + {\text{ HO}}_{2}^{*}$$

13 $${\text{O}}_{{3}} + {\text{HO}}_{2}^{*} \to {\text{2 O}}_{{2}} + {\text{HO}}*$$

14 $${2}\;{\text{HO}}_{{2}} * \to {\text{O}}_{{2}} + {\text{H}}_{{2}} {\text{O}}_{{2}}$$

15 $${\text{HNO}}_{{{2}({\text{l}})}} \rightleftharpoons {\text{NO}}_{2(1)}^{ - } + {\text{ H}}_{(1)}^{ + }$$

16 $${\text{HNO}}_{{{3}({\text{l}})}} \rightleftharpoons {\text{NO}}_{3(1)}^{ - } + {\text{H}}_{(1)}^{ + }$$

17 $${\text{2NO}}_{{{2}({\text{l}})}} + {\text{H}}_{{2}} {\text{O}}_{{({\text{l}})}} \to {\text{NO}}_{2}^{ - } + {\text{NO}}_{3(1)}^{ - } + {\text{2H}}_{(1)}^{ + }$$

18 $${\text{NO}}_{{({\text{l}})}} + {\text{NO}}_{{{2}({\text{l}})}} + {\text{H}}_{{2}} {\text{O}}_{{({\text{l}})}} \to {\text{2NO}}_{2(1)}^{ - } + {\text{2H}}_{(1)}^{ + }$$

19 $${\text{O}}_{{{3}({\text{l}})}} + {\text{NO}}_{2(1)}^{ - } \to {\text{NO}}_{{{2}({\text{l}})}} + {\text{NO}}_{3(1)}^{ - }$$

From H_2_O_2_ of (14),

20 $${\text{NO}}_{2}^{ - } + {\text{H}}_{{2}} {\text{O}}_{{{2}({\text{l}})}} \to {\text{ONOO}}_{(1)}^{ - } + {\text{H}}_{{2}} {\text{O}}_{{({\text{l}})}}$$

21 $${2}\;{\text{NO}}_{{{2}({\text{g}})}} \rightleftharpoons {\text{HONO}}_{{({\text{aq}})}} + {\text{H}}^{ + } + {\text{NO}}_{3}^{ - }$$

22 $${\text{HONO}}_{{({\text{aq}})}} \rightleftharpoons {\text{H}}^{ + } + {\text{NO}}_{2}^{ - }$$

23 $${\text{HONO}}_{{({\text{aq}})}} \rightleftharpoons {\text{HONO}}_{{({\text{g}})}}$$

### Evaluation of material compatibility

The sterilization process exposed the sample tubes to active species in the air plasma such as atomic oxygen and nitrogen oxides. Therefore, there is a need to confirm the material compatibility of these reactive species. No visual degradation owing to sterilization treatment was observed in any material; hence, the chemical bonding of the material surface was also investigated using FTIR spectroscopy. Treatment of PET with active species in the air plasma resulted in maximum changes, including a C–H change of -6.8% for the benzene ring (722 cm^−1^) and + 18% for the C–H (methylene group, 2967 cm^−1^) after 2–8 h of treatment. After 10 h the percentages of C–O–C (+ 2.6%) and C=O (+ 8.2%) increased with oxidation. Treatment of PVC resulted in decreases of C–Cl and C–H after 2–10 h of treatment. The maximum changes were for C–Cl: − 12% and C–H: − 17%. A C=O feature showed a maximum decrease of − 21% after 6 h treatment and an increase of + 3.8% owing to oxidation after 8-h treatment. Treatment of silicone tube resulted in changes within 5% for all components [C–H, Si–O–Si, Si–C (786 cm^−1^), Si–C (1258 cm^−1^)] except for C–H, which increased by + 5.8% after the 2 h treatment.

### Investigation of spore inactivation mechanism

In order to investigate the inactivation mechanism of the bacterial spore in long narrow tube using oxygen and air plasmas produced by the atmospheric barrier discharge. One of the possibilities of the inactivation mechanism is destruction of the surface of the bacterial spore^33–35^. Therefore, decomposition characteristics of the material of the bacterial spore coat have been investigated. The major composition of the spore coat of bacterial spore is keratin, aspartic acid, glutamic acid and dipicolinic acid^35,36^. In this experiment, the chemical composition of these materials after irradiation with the oxygen or air plasma were analyzed using the IR spectra.

### Keratin

**Figure 6:**
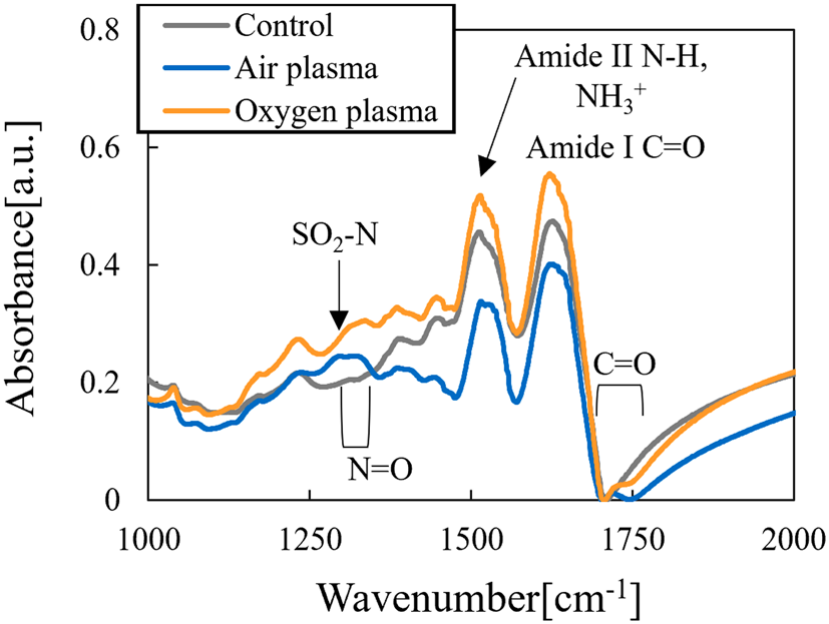
FTIR spectrum of wool keratin treated with active species.

**Figure 7:**
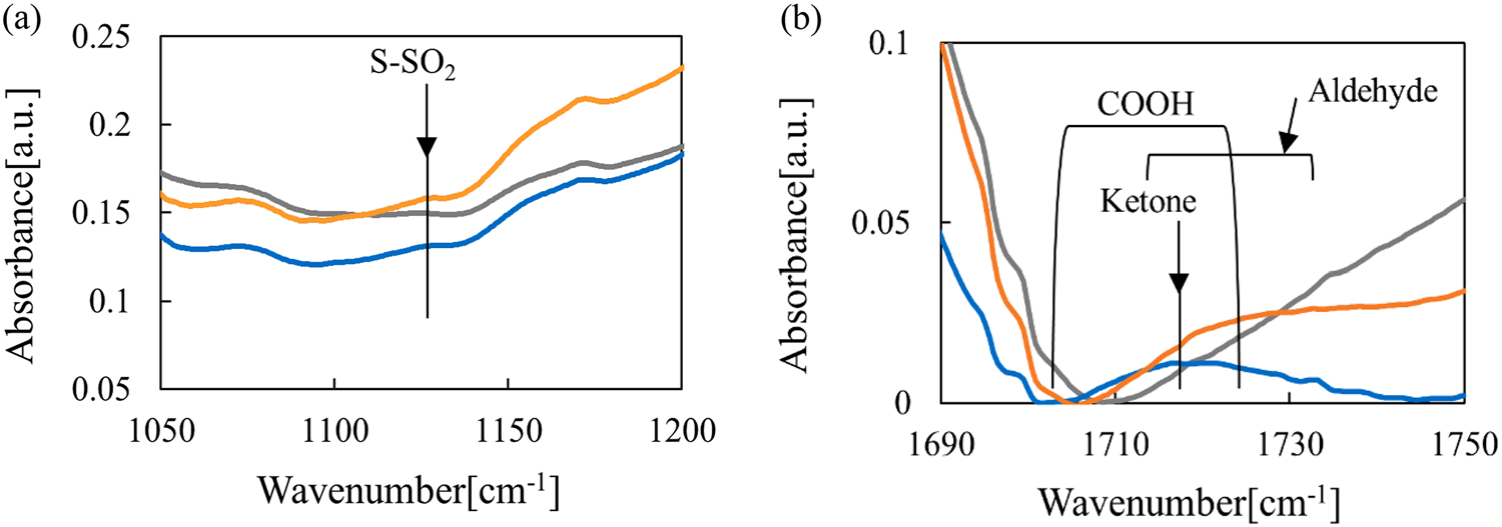
(**a**) FTIR spectrum of wool keratin treated with active species over the ranges of 1050–1200 cm^−1^ and (**b**) in 1690–1750 cm^−1^.

Figure 6 shows FTIR spectra of keratin treated with active species from the air plasma and oxygen plasma. Active species from both air and oxygen plasmas generated SO_2_ and N=O dimers in keratin samples. In spectra of the air plasma treated samples, there were decreases in amide and NH_3_^+^ peaks from salt bridges (i.e., two amino acids forming an ionic complex through COOH/COO^−^ and NH_2_/NH_3_^+^ interactions. Thus, salt bridges were oxidized, and the number of these bonds decreased, which may have reduced the rigidity of keratin. The peaks at 1290–1320 cm^−1^ can be assigned to SO_2_-N and N=O. Figure 7a,b show an expanded view of the results shown in Fig. 6. Both air and oxygen plasmas produced SO_2_, CHO, and ketone (C=O) features confirming the oxidating action of ozone. In the case of the air plasma, HNO_3_ might also contribute to oxidation of keratin.

### Aspartic acid

**Figure 8:**
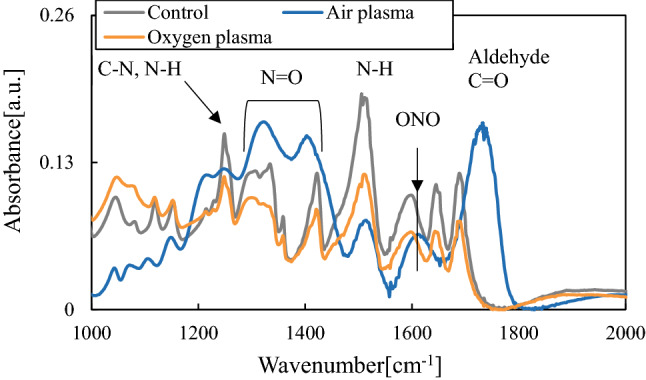
FTIR spectra of aspartic acid treated with active species.

Figure 8 shows FTIR spectra of aspartic acid treated with active species from air plasma and oxygen plasma. Following oxy gen plasma treatment, the FTIR results were similar to those of the untreated sample. The peaks around the N–H region were slightly decreased owing to oxidization by ozone. Conversely, for the sample treated by the air plasma, the spectrum was markedly different from that of the untreated sample, and new features appeared from aldehyde-derivatives C=O, nitrite (ONO), and N=O whereas C–H and N–H peaks were reduced. The results for the air and oxygen plasmas, indicate that aspartic acid was particularly susceptible to denaturation from nitrogen oxides derived from the air plasma. Thus, it was likely that the C–H and N–H were oxidized to C=O and N=O. Additionally, a new C=O peak generated by oxidation was observed in the results for the air plasma only. These results confirmed that the combined oxidation capacity of NO_x_ produced by air plasma and active species, such as ONOO^−^ formed secondarily from NO_x_ added to the ozone oxidation of oxygen plasma. Thus, the highly effective sterilization effected by the air plasma maybe attributed to the compositional denaturation of organic materials through oxidation mediated by NO_x_ and active species generated between ozone and NO_x_.

### Glutamic acid

**Figure 9:**
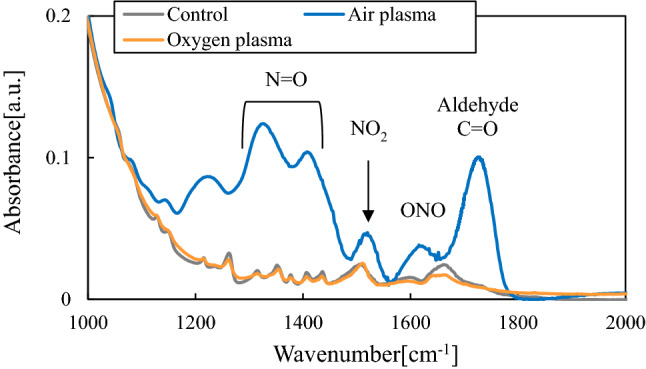
FTIR spectra of glutamic acid treated with active species.

Figure 9 shows the FTIR spectrum of glutamic acid treated with active species generated from air plasma and oxygen plasma. The peak locations of untreated samples and those subjected to the oxygen plasma were almost identical. The results for glutamic acid and aspartic acid were similar owing to their similar structures. Thus, the effects of ozone from the oxygen plasma on glutamic acid were limited. Conversely, in the case of the air plasma, N=O and aldehyde-derived C=O peaks were identified. These features are attributed to oxidation of NH_2_ and C-H.

### Dipicolinic acid

**Figure 10:**
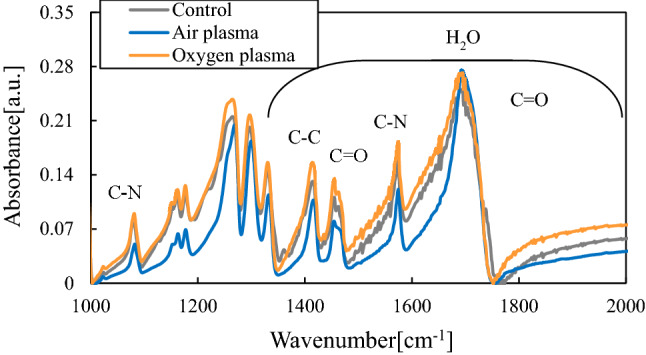
FTIR spectra of dipicolinic acid treated with active species.

Figure 10 showed the FTIR spectra of dipicolinic acid treated with active particles derived from air and oxygen plasma. There was little difference in the spectral peaks among the untreated samples, and those subjected to air and oxygen plasmas. Water vapor peaks appeared at 1300–2000 cm^−1^ in the untreated samples and those subjected to the oxygen plasma; however, these peaks were attributed to external contamination associated with the instruments. Because dipicolinic acid was not denatured by the sterilization treatment, dipicolinic acid may not be related to the disinfection processes directly.

## Conclusions

Using a DBD torch plasma source with oxygen gas and air, the inside of a tube 4 mm in diameter and 60 cm in length was sterilized in 30 min. Ozone and NO_x_ were identified by FTIR and HNO_3_ and N_2_O_5_ were found to be effective active species for sterilization of long narrow tubing. The sterilization method in this study effectively sterilized silicone tubing with minimal material degradation. The results of active species treatment of organic materials (keratin, aspartic acid, glutamic acid, dipicolinic acid) comprising bacterial spores were as follows. Keratin showed chemical composition changes from both ozone only from oxygen plasma and ozone and NOx from air plasma treatments. For other organic materials, ozone induced minimal change in the composition, whereas ozone and NO_x_ changed the compositions of aspartic acid and glutamic acid. These results suggest that a high sterilization performance of air plasma-derived active gases maybe attributed to strong denaturing effects on the chemical composition of organic materials by the mixture gases of ozone and NO_x_.

## Data Availability

The datasets used and analyzed during the current study are available from the corresponding author on reasonable request.

## References

[1] Shintani H (2017). Ethylene oxide gas sterilization of medical devices. Biocontrol Sci..

[2] Hayashi N, Guan W, Tsutsui S, Tomari T, Hanada Y (2006). Sterilization of medical equipment using radicals produced by oxygen/water vapor RF plasma. Jpn. J. Appl. Phys..

[3] Nagatsu M (2007). Sterilization characteristics inside narrow tube using nitrogen oxides generated by atmospheric pressure air plasma. J. Plasma Fusion Res..

[4] Manzoor N, Qasim I, Khan I, Ahmed W, Guedri K, Bafakeeh OT, Tag-Eldin E, Galal A (2022). Antibacterial applications of low-pressure plasma on degradation of multidrug resistant *V. cholera*. Appl. Sci..

[5] Mastanaiah N, Johnson JA, Roy S (2013). Effect of dielectric and liquid on plasma sterilization using dielectric barrier discharge plasma. PLoS ONE.

[6] Choudhury B, Revazishvili T, Lozada M, Roy S, Mastro EN, Portugal S, Roy S (2023). Distributed compact plasma reactor decontamination for planetary protection in space missions. Sci. Rep..

[7] Sato T, Furuya O, Ikeda K, Nakatani T (2008). Generation and transportation mechanisms of chemically active species by dielectric barrier discharge in a tube for catheter sterilization. Plasma Process. Polym..

[8] Sato T, Furuya O, Nakatani T (2009). Characteristics of nonequilibrium plasma flow and its sterilization efficacy in a tube at atmospheric pressure. IEEE Trans. Ind. Appl..

[9] Yonesu A, Hara K, Nishikawa T, Hayashi N (2016). Characteristics of surface sterilization using electron cyclotron resonance plasma. Jpn. J. Appl. Phys..

[10] Kitazaki S, Tanaka A, Hayashi N (2014). Sterilization of narrow tube inner surface using discharge plasma, ozone, and UV light irradiation. Vacuum.

[11] Portugal S, Roy S, Lin J (2017). Functional relationship between material property, applied frequency and ozone generation for surface dielectric barrier discharges in atmospheric air. Sci. Rep..

[12] Ono R, Oda T (2006). Ozone production process in pulsed positive dielectric barrier discharge. J. Phys. D: Appl. Phys..

[13] Ahn HS, Hayashi N, Ihara S, Yamabe C (2003). Ozone generation characteristics by superimposed discharge in oxygen-fed ozonizer. Jpn. J. Appl. Phys..

[14] Anderson JG, Kaufman F (1972). Kinetics of the reaction OH (v = 0) + O_3_ → HO_2_ + O_2_. Chem. Phys. Lett..

[15] Atkinson R, Baulch DL, Cox RA, Hampson RF, Kerr JA, Kerr JA (1989). Evaluated kinetic and photochemical data for atmospheric chemistry: supplement III. IUPAC subcommittee on gas kinetic data evaluation for atmospheric chemistry. J. Phys. Chem..

[16] Falkenstein Z, Coogan JJ (1997). Microdischarge behaviour in the silent discharge of nitrogen–oxygen and water–air mixtures. J. Phys. D: Appl. Phys..

[17] Hayashi N, Inoue Y, Kyumoto Y, Kukita T (2020). Characteristics of differentiation of osteoclast cells irradiated with active species in atmospheric oxygen plasma. Jpn. J. Appl. Phys..

[18] Lilley AJ, Roy S, Michels L (2022). Performance recovery of plasma actuators in wet conditions. J. Phys. D Appl. Phys..

[19] Ricchiuto AC, Borghi CA, Cristofolini A, Neretti G (2020). Measurement of the charge distribution deposited on a target surface by an annular plasma synthetic jet actuator: Influence of humidity and electric field. J. Electrostat..

[20] Algie JE, Lindsay JA (1983). Some physical properties of coat material from Bacillus stearothermophilus spores. Curr. Microbiol..

[21] Kadota H, Iijima K, Uchida A (1965). The presence of keratin-like substance in spore coat of Bacillus subtilis. Agric. Biol. Chem..

[22] Remy A, De Geyter N, Reniers F (2023). Interplay between nitrogen oxides and ozone in a filamentary dielectric barrier discharge at various frequencies. Plasma Process Polym..

[23] Sato T, Fujioka K, Ramasamy R, Urayama T, Fujii S (2006). Sterilization efficacy of a coaxial microwave plasma flow at atmospheric pressure. IEEE Trans. Ind. Appl..

[24] Nakamuraya, K., Yoshino, D., Nakajima, T. & Sato, T. Development of sterilization method by air plasma. *Proc. 45th Autumn Meet of Jpn Soc. Mech. Eng. Tohoku Branch*, **113**, 25–26 (2013).

[25] Tamaoki M (1983). Sources, Sinks and Residence Time of Atmospheric N_2_O. J. Environ. Conserv. Eng..

[26] Kosugi N, Imai H, Matsumoto J, Kato A, Kajii Y (2005). Development of an NO3/N205 analyzer utilizing a laser-induced-fluorescence technique and evaluation of winter nocturnal oxidation by nitrogen oxides. J. Jpn. Soc. Atmos. Environ..

[27] Sotelo JL, Beltran FJ, Benitez FJ, Beltran-Heredia J (1987). Ozone decomposition in water: Kinetic study. Ind. Eng. Chem. Res..

[28] Muramatsu K, Sato T, Nkajima T, Nagasawa T, Nakatani T, Fujimura S (2020). Sterilization in liquids by air plasma under intermittent discharge. Mech. Eng. J..

[29] Spicer CW, Kenny DV, Ward GF, Billick IH (1993). Transformations, lifetimes, and sources of NO_2_, HONO, and HNO_3_ in indoor environments. Air Waste.

[30] Sato T, Ueda Y, Takahashi K, Takaki KJ (2021). J. Multiphase Flow.

[31] Akaike T (2015). Host defense and oxidative stress signaling in bacterial infection. Jpn. J. Bacteriol..

[32] Ogata Y, Tabushi I (1957). The Organic Oxidation by Nitrogen Compounds. J. Synth. Org. Chem Jpn..

[33] Moreau S (2000). Using the flowing afterglow of a plasma to inactivate Bacillus subtilis spores: Influence of the operating conditions. J. Appl. Phys..

[34] Hury S, Vidal DR, Desor F, Pelletier J, Lagarde T (1988). A parametric study of the destruction efficiency of Bacillus spores in low pressure oxygen-based plasmas Get access Arrow. Lett. Appl. Microbiol..

[35] Roth S, Feichtinger J, Hertel C (2010). Characterization of Bacillus subtilis spore inactivation in low-pressure, low-temperature gas plasma sterilization processes. J. Appl. Microbiol..

[36] Hayashi N, Kometani R, Yoshida Y (2013). Treatment of dipicolinic acid and inactivation mechanism of thermophile spores using active oxygen. Jpn. J. Appl. Phys..

